# Progress in Bacteriology

**Published:** 1901-05-25

**Authors:** 


### Progress in Bacteriology.

**Cystitis.**—Brown[1] has investigated the bacteriology of a hundred consecutive cases of cystitis. He points out that with hyperacidity of the urine all the symptoms of cystitis may be simulated, even to the appearance of pus, blood, and mucus, but that in these cases the urine is, of course, sterile. In cases where there is doubt as to whether the kidney or the bladder is the seat of infection, attention should be paid to the specific gravity, the percentage of urea, and the nature of the blood cells. A low specific gravity, large amount of albumen, diminished urea, crenated red corpuscles, all point to kidney infection. Suprapubic puncture of the bladder may be done with impunity, and the most reliable specimens are obtained in this way. Of the twenty-six acute cases investigated the incubation period varied from three to twenty days. The following organisms were found:—B. coli, fifteen times; staphylococcus pyogenes albus, five; staphylococcus pyogenes aureus, twice; B. pyocyaneus, once; B. typhosus, once; Proteus vulgaris, once. The urine was acid except when cocci and Proteus were present, and hyperacid with B. coli and typhosus. In the thirty-one chronic cases the same organisms were found, the B. coli being present in sixteen, in one of which it was associated with the tubercle bacillus. This was the only double infection. In only six of the thirty-one cases was the urine alkaline. Of the six tubercular cases all apparently started in the bladder, and except in the instance recorded above the infection was always a pure one. The tubercular cystitis was characterised by the presence of large quantities of blood. Some interesting cases of pyelitis and of pyelonephritis are also recorded. Of the two acute cases, in one the urine was acid and the exciting organism the B. coli. In the other the organism present was the Proteus vulgaris and the urine was alkaline. Half of the twelve chronic cases were caused by the B. coli, and the remainder by Proteus vulgaris and staphylococci. Eight were associated with cystitis. Six cases of pyelonephritis were tubercular, and of these four had been infected from the bladder. Young, in discussing the paper, stated that the B. coli was less frequently the exciting cause of cystitis in males than in females. He had also met with cases due to B. lactis ærogenes, gonococcus, Proteus Zenkeri, and streptococci. The Proteus Zenkeri was present in an acid urine.

**Diphtheria.**—The great practical difficulty in the bacteriological diagnosis of diphtheria is that the pseudo-diphtheria bacillus of Hoffman is likely to be mistaken for the true organism. Cobbett,[2] during a recent epidemic of diphtheria at Cambridge and Chesterton, made 950 examinations from the throats of 650 persons who were at the time in apparently good health. The true Klebs-Löffler bacillus was present in 19 only, whilst Hoffman's bacillus was found in 157. Cobbett claims that the Hoffman bacillus can be distinguished on morphological evidence, quite apart from cultural characteristics. Thus he recognises four main types of the true diphtheria bacillus. (1) Long, faintly-stained irregularly beaded bacilli. (2) Regularly beaded bacilli resembling streptococci, (3) segmented baccilli, (4) uniformly-stained organisms. The Hoffman pseudo-bacillus differs from the foregoing in being more oval in shape, in having only one or at most two unstained septa, and in being shorter and stouter. The pseudo-bacillus does not form acid in litmus glucose media, nor does it give rise to local œdema when injected into animals. Bacilli of this class are constantly present in the noses and throats of children of the poorer class, whether they have been in contact with cases of diphtheria or not. The bacilli are quite harmless, and the author, unlike other authorities, does not believe that there is any evidence to prove that they can be converted into virulent diphtheria organisms. There can be no doubt that confusion between these two organisms has done much to bring discredit upon the bacteriological diagnosis of the disease, and it is sincerely to be hoped that this article will prove of service to future investigators. Cobbett suggests that in this country as in some American cities it should be made a rule that no case of diphtheria is discharged from isolation until two or three negative cultures have been obtained.

**Atrophic Rhinitis and Ozæna.**—Stein[3] reviews the bacteriology of this disease, and adds details of 51 cases which he has examined. The following organisms have been associated with atrophic rhinitis: a diplococcus or short bacillus, the bacillus mucosus described by Löwenberg and Abel; a similar organism setting up decomposition with offensive odour, described by Hajek; the gonococcus (Stork); a bacillus resembling the diphtheria bacillus, described by Belfante and Della Vedova; a putrefactive bacillus described by Perez. Stein concludes that the true causative agent of the disease is the B. mucosus, and that the ozæna is a complication consequent upon secondary infection by putrefactive organisms. The diphtheria-like organism was present in 26 out of 51 cases; it has no pathogenic properties, and is also present in some healthy throats and noses. The bacillus mucosus was found in 44 cases in cultures, in three cases on fresh films only, and in the remaining four cases involution forms were found in two, whilst two were negative.

**Vaccinia.**—Copeman,[4] continuing his researches on the bacteriology of vaccinia and variola, states that he finds the method of cultivation in eggs very uncertain, but that better results are obtained if the eggs used are fertilised, as shown by the commencing development of the embryo after incubation. Still more reliable results are obtained by using collodion capsules filled with beef broth and inoculated with a trace of vaccine lymph free from extraneous organisms. These capsules are introduced into the peritoneal cavities of rabbits, and kept there for about a fortnight so as to permit the body lymph to dialyse through the capsule walls and nourish the bacteria. In this manner he has obtained a resting stage of the specific bacillus, which is found in zooglea masses made up of bodies resembling spores. These spores were, when inoculated into calves, found to be capable of producing a typical eruption of vaccinia. It is obvious that these spore-like bodies may have minute quantities of vaccine lymph around them, and to this lymph, and not to the spores, the injections may owe their success. The same criticism applies to recent results recorded by Fünck.[5] He maintains that the specific organism of vaccinia and variola is a protozoon. This can be demonstrated in fresh specimens of lymph or in stained films, a magnification of 500 diameters being sufficient for the purpose. The parasite is a motile sphere, measuring from 2 $\mu$ to 10 $\mu$, of greenish colour; it sometimes forms raspberry-like bodies (20 $\mu$ by 30 $\mu$), which are cysts, full of spores. These morulas he has succeeded in isolating, and emulsions prepared from them if injected into susceptible animals give rise to all the classical symptoms of vaccinia, and the animals are rendered refractory to subsequent inoculations of vaccine. The variolous pustule contains a protozoon morphologically similar to that in the vaccine. We have already mentioned the objection which may be made to these results, but the resemblance between the raspberry masses found by Fünck and the zooglea masses of spores described by Copeman is worthy of notice.

**Micro-organisms in Normal Tissues.**—In order to determine whether bacteria were present in normal healthy organs, Ford [6] adopted the following procedure: Healthy animals in good condition were suddenly killed, the organs removed under the strictest antiseptic precautions and placed in specially adapted vessels of nutrient medium in which they were broken up by means of sterilised glass rods. In the case of rabbits, cultures from the organs showed growth in sixty-six per cent. of the cases, the organisms found in gelatine being the staphylococcus albus, B. coli, B. proteus, and B. paracolon. In both the staphylococcus aureus, B. proteus, B. mesentericus were also found. With guinea-pigs sixty-one per cent. of the cultures showed growth; dogs, eighty-three per cent.; with cats, seventy-seven per cent., including colonies of B. megatherium, B. zopfii, B. mycoides, and two unknown spore-bearing varieties of bacteria. In no case did growth appear until the fourth day, and in some it was delayed until the seventeenth day, so that the author thinks that the conflicting results of former observers may be due to the fact that too short a period of incubation was given. It is noteworthy that each animal possessed its distinct bacteriology, and that organisms were found to select certain organs to the exclusion of others.

[1] Bullet. Johns Hopk. Hosp., Jan. 1., 1901. [2] Jour. of Hygiene, Vol. I., No. 2, April, 1901. [3] Central. f. Bakt., Dec. 15, 1900. [4] Brit. Med. Jour., Feb. 23, 1901. [5] Ibid. [6] Jour. of Hygiene, Vol. I., No. 2, April, 1901.

